# Fourteen-Year Analysis of Percutaneous Nephrolithotripsy Outcomes: Evolution of Technique and Future Perspectives

**DOI:** 10.1590/S1677-5538.IBJU.2025.0403

**Published:** 2025-08-30

**Authors:** Fabio C. Vicentini, Carlos A. Batagello, Giovanni S. Marchini, Fabio C. M. Torricelli, Artur Brito, Alexandre Danilovic, Guilherme Gentile, Henrique Lepine, Priscila Kuriki Mota, Daniel Beltrame Ferreira, Rodrigo Perrella, David Cohen, Claudio B. Murta, Valter Cassao, Joaquim A. Claro, William Nahas, Eduardo Mazzucchi

**Affiliations:** 1 Faculdade de Medicina da Universidade de São Paulo Divisão de Urologia São Paulo SP Brasil Divisão de Urologia, Faculdade de Medicina da Universidade de São Paulo - FMUSP, São Paulo, SP, Brasil; 2 Hospital Israelita Albert Einstein Divisão de Urologia São Paulo SP Brasil Divisão de Urologia, Hospital Israelita Albert Einstein, São Paulo, SP, Brasil; 3 Hospital Brigadeiro Divisão de Urologia São Paulo SP Brasil Divisão de Urologia, Hospital Brigadeiro, São Paulo, SP, Brasil; 4 Faculdade de Medicina da Universidade de São Paulo São Paulo SP Brasil Faculdade de Medicina da Universidade de São Paulo – FMUSP, São Paulo, SP, Brasil

**Keywords:** Kidney Calculi, Nephrolithotomy, Percutaneous, General Surgery

## Abstract

**Purpose:**

The treatment of kidney stones has undergone continuous evolution. Despite the evolution of retrograde intrarenal surgery, percutaneous nephrolithotomy (PNL) remains the gold standard treatment for large and complex stones. We aimed to evaluate and analyze the temporal evolution of the results of the PNLs conducted at two teaching hospitals.

**Results:**

Data from 2660 patients were studied between 2009 and 2022. The rate of complex stones (Guy's 3 and 4) was 55.3%. Supine position was used in 82.1% of the cases. In 74.7% of cases, only 1 puncture was performed. The median surgery time was 120 min (15-240 min). The overall complication rate was 12.2%, the transfusion rate was 4.5%, and the success rate was 59.8%. Regarding temporal evolution, the use of the supine position increased from 73% in 2009 to 100% in 2022 (p < 0.001). The use of nephrostomy dropped from 81.8% to 26.5% (p<0.001), the median duration of surgery dropped from 145 to 130 min, the median time of use of fluoroscopy went from 12 to 8 min, the rate of blood transfusions dropped from 11.5% to 2.8% (p= 0.009), and the complication rate dropped from 18.2% to 12% (p= 0.002), while the average length of stay dropped from 81.8 hours to 50 hours.

**Conclusions:**

PNL is an effective surgical option for treating complex kidney stones. The implementation of several technical aspects, along with the standardization of the procedure, led to a significant improvement in most outcomes, reducing complication rates but leaving room for technique improvements in terms of success rates.

## INTRODUCTION

Despite the recent evolution of the retrograde intrarenal surgery technique (RIRS), percutaneous nephrolithotomy (PNL) remains the gold standard for complex and large kidney stones (1). The ability to break and aspirate a large stone burden in a low-pressure environment and more quickly makes PNL the preferred treatment method for these types of calculi. In regions where large or staghorn stones are common, PNL will continue to be used for a long time. Even in the United States, its use has been increasing recently, indicating that PNL still needs further study and development (2).

**Figure 1:**
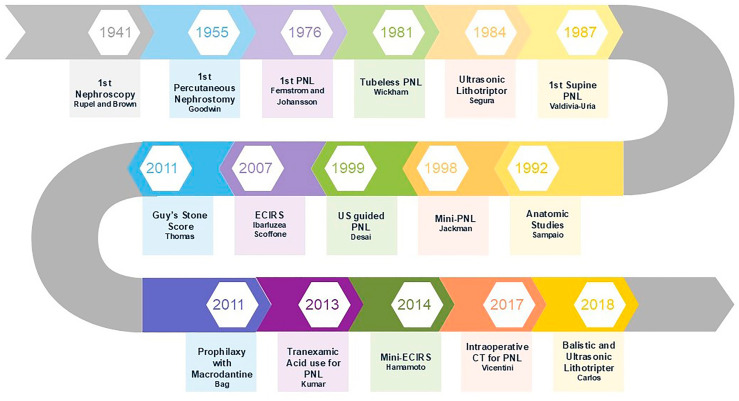
Timeline of innovations that led to the development and evolution of PCNL.

Many developments have occurred since the first percutaneous kidney stone extraction by Fernstrom and Johansson in 1976 (3). These procedures were initially performed with patients in the prone position (PRO) and under radiological guidance, which has become the standard position ever since. Percutaneous nephrolithotomy was developed and widely adopted through this approach, especially due to the work of Drs. Arthur Smith, Joseph Segura, and Ralph Clayman, the founding members of the Endourological Society, who proved that PRO is an effective position (4). Over time, some limitations of PRO became apparent, leading to the need for improvements. In 1987, Dr. José Gabriel Valdivia-Uría described PNL in the supine position (SUP) (5), publishing a series of more than 500 cases a few years later (6). This evolution eventually resulted in the development of the Galdakao-Valdivia modified positioning for endoscopic combined intrarenal surgery (ECIRS) (7), which has been shown to enhance PNL outcomes (8). In Figure-1, we see the timeline of some of the major developments in PNL, illustrating that it remains a work in progress.

There are numerous PNL series in the literature that present their outcomes; however, there is a scarcity of studies evaluating their evolution over time (9).

The purpose of this study is to evaluate the surgical outcomes of PNL at two major institutions and to examine the temporal evolution of these outcomes.

## MATERIALS AND METHODS

This retrospective study involved collecting data from the electronic medical records of the Urology Department at Hospital das Clínicas, University of São Paulo Medical School (HC), focusing on patients who underwent PNL from 2010 to December 2022. Concurrently, data were also collected from the prospective database of percutaneous nephrolithotomies performed at Hospital Brigadeiro (HB), a public Hospital in São Paulo, with a consistent program of residency in Urology, during the same period. We conducted a temporal analysis to evaluate and compare the annual results. The data from the two departments were analyzed both independently and collectively when they were comparable. There was no direct contact with patients; only data from electronic medical records were used. This study protocol was submitted for review by CAPPESQ and registered on the Brazil Platform under CAAE 72842723.7.0000.0068.

### Inclusion criteria

Patients who underwent PNL at HC and HB, with analyzable data in the electronic medical records of the Urology Division of HC and the prospective database of HB, respectively, from 2009 to 2022, were included in the study.

### Exclusion Criteria

Patients with incomplete or unanalyzable data were excluded.

The variables evaluated included demographic data (sex, age, weight, and height); renal calculus data (size, density, and classification according to Guy's Stone Score (10); patient positioning on the surgical table, which varied between supine or prone position (supine variations were considered as a group); surgery time; fluoroscopy usage time; nephrostomy usage rate; blood transfusion rate; rate of stone-free patients at the end of surgery (absence of stones at the end of surgery, based on endoscopic and radioscopic evaluation); success rate, defined as the absence of stones > 4 mm in the first postoperative day CT scan; and complication occurrence rate.

### Database Characteristics

For HC, a database covering the period from 2010 to 2022 with 38 variables was used. The cleaning and categorization of the selected variables were carried out according to the objectives of the analysis. For HB, four databases were used: three databases from 2009 to 2016 (two with 32 variables and one with 78 variables) and one database from the period 2018 to 2022 (69 variables). Initially, the common variables across all databases were identified. Subsequently, the selection, cleaning, and organization of the variables for analysis were conducted, and finally, all the databases were merged, resulting in a single comprehensive database.

### Statistical analysis

For the statistical analysis, an initial descriptive analysis of the data was conducted, which included estimates of the mean, median, standard deviation, 25th and 75th percentiles, interquartile range, and the minimum and maximum of the quantitative variables, as well as simple and relative frequencies of the qualitative variables. To assess the association between the qualitative variables, the chi-square test was employed. The Mann-Whitney U test was used to determine the difference between the two groups, due to the non-normality of the analyzed variable, as verified by the Shapiro-Wilk test. A significant level of 5% was used, and all analyses were performed in the R 4.1.0 environment (R Core Team, 2021).

## RESULTS

A total of 2,660 PNL patients were analyzed from 2009 to 2022, with 1,452 patients at HC and 1,208 at HB. Table-1 presents the demographic data. We observed that the rate of cases considered complex (Guy's 3 and 4) was 55.3%.

**Table 1:** Frequencies of the variables age, BMI, sex, ASA, maximum diameter, and Guy's Stone Score.

Variable		N	%
Age Median (min-max)	51 (7-90)	2656	
BMI Median (min-max)	27.3 (16-58.1)	2440	
Sex N= 2660	Female	1624	61.0
	Male	1036	39.0
ASA N= 2047	1	695	34.0
	2	1175	57.4
	3	174	8.5
	4	3	0.1
Maximum diameter N= 2408	5 - 10 mm	141	5.8
	11 - 15 mm	294	12.2
	16 - 20 mm	531	22.1
	21 - 25 mm	416	17.3
	26 - 30 mm	344	14.3
	31 - 35 mm	160	6.6
	36 - 40 mm	203	8.4
	> 40 mm	319	13.3
Guys N= 2169	I	354	16.3
	II	615	28.4
	III	818	37.7
	IV	382	17.6

N = number of patients; MD = median; Min = minimum; Max= Max; % = relative frequency. BMI = body mass index

In relation to the data concerning the perioperative period, the supine position was utilized in 82.1% of the cases. In 74.7% of the cases, the surgeries were conducted with only one renal puncture. The median duration of the surgeries was 120 minutes, with a range from 15 to 420 minutes. Other parameters are shown in Table-2.

**Table 2:** Intraoperative data at each institution and the total sum.

Variables		HC	HB	Total
**Positioning %(n)**		N= 969	N= 1207	N= 2176
	Supine	72.8% (705)	89.6% (1082)	82.1% (1787)
	Prone	27.2% (264)	10.4% (125)	17.9% (389)
**Number of punctures %(n)**		**N= 747**	**N= 1204**	**N= 1951**
	1	73.5% (549)	75.6% (910)	74.7% (1459)
	2	23.5% (175)	19.7% (237)	21.1% (412)
	3	2.8% (21)	3.8% (46)	3.4% (67)
	4	0	0.6% (7)	0.4% (7)
	5	0.1% (1)	0.3% (4)	0.3% (5)
	7	0.1% (1)	0	0.1% (1)
**MD Surgery Time (IQR)**		**120 (60)**	**105 (70)**	**120 (64,5)**
**Use of flexible nephroscope %(n)**		N= 302	N= 1184	N= 1486
	Yes	92.4% (279)	14.2% (168)	30.0% (447)
**Nephrostomy tube %(n)**		**N= 1316**	**N= 1126**	**N=2442**
	Yes	65.3% (859)	77.1% (868)	70.5% (1709)
**Fluoroscopy use time MD(IIQ)**		9 (9)	12 (11)	11 (10)
**Intraoperative Stone free %(n)**		**N= 922**	**N= 1188**	**N= 2210**
	Yes	65.8% (607)	63.8% (758)	64.7% (1365)

The rate of perioperative and postoperative complications was 12.2%, while the transfusion rate was 4.5%.

The overall success rate was 59.8%, based on the absence of fragments larger than 4 mm on the CT scan on the first postoperative day. The success rates during the period in both services, according to the Guy's Stone Score, were 85.7%, 63.7%, 43%, and 28.2% for Guy's 1, 2, 3, and 4 cases, respectively (p<0.001).

Regarding the temporal analysis, we observed that in 2010, 73% of the patients were operated on in the supine position, evolving to 100% in 2022 (p<0.001).

The use of nephrostomy at the end of surgery fell from 81.8% in 2009 to 26.5% in 2022 (p<0.001).

The median surgery time ranged from 145 (IQR 65) minutes in 2009 to 130 (IQR 70) minutes in 2022 (p< 0.001), as well as a decrease in the time of fluoroscopy use, from 12 (IQR 8) in 2009 to 8 (IQR 10.8) minutes in 2022 (p<0.001).

The joint analysis of transfusion rates shows a decrease from 11.5% in 2010 to 2.8% in 2022 (p=0.009), as well as total complication rates from 18.2% in 2009 to 12% in 2022 (p=0.002).

The percentage of hospitalizations lasting up to 4 days increased from 82% in 2009 to 92.1% in 2022 at HC (p<0.001). At HB, the average length of hospital stay decreased from 81.8 hours (SD± 69.4) in 2009 to 50 hours (SD± 25.3) in 2022.

## DISCUSSION

Between 2009 and 2022, we analyzed a total of 2,660 PNL cases. Due to the retrospective nature of the study, we encountered limitations inherent to this method in data collection, which posed challenges to the analysis. However, we successfully conducted a temporal evaluation, achieving clarity on certain specific aspects of the surgery.

Regarding perioperative aspects, 82.1% of surgeries were performed with the patient in supine position. Over the years, the supine position has become the standard for PNL in both institutions. In 2010, 73% of patients were operated on in the supine position, which increased to 100% by 2022 (p<0.001). Bart's flank-free modified position (11) was established as the standard position after several tests with other positions, as it proved to be the most effective for PNL as well as for endoscopic combined intrarenal surgery (ECIRS) in our experience (12). A randomized study by Perrella et al. demonstrated that positioning does not affect success rates, even for complex stones. However, the supine position is associated with a 30-minute reduction in surgery time and a lower likelihood of severe complications (13), findings that were confirmed in a recent meta-analysis (14). As previously shown, practitioners who initially perform PNL in the prone position and switch to the supine position tend to predominantly use the supine position over time (15). This is because supine PNL is faster, involves less patient manipulation and draping, facilitates ECIRS performance, offers the same success rate for all types of cases, and potentially reduces the risk of complications in complex cases (16). It represents a point of no return.

The global rate of nephrostomy use at the end of surgery was 70.5%, demonstrating a significant decrease from 81.8% in 2009 to 26.5% in 2022 (p<0.001). Historically, a nephrostomy tube was routinely left after PNL, a practice reported by Clayman et al. (17). Interestingly, during the same period, Wickham et al. (18) documented 250 cases managed without routine nephrostomy, recommending its use only for instances of severe bleeding or large perforations. At that time, PNL was performed without any ureteral drainage, as ureteral stents had not yet been introduced, making nephrostomy the logical choice for kidney drainage. However, since a 2010 meta-analysis by Borges et al., which included 10 randomized studies, it was demonstrated that the "tubeless" procedure was as safe as leaving a nephrostomy tube in uncomplicated cases, with the added benefits of reduced patient pain, shorter hospital stays, and decreased urinary leakage time (19). Our observations further support that omitting the nephrostomy tube is associated with less pain and a one-day reduction in inpatient stay. Surprisingly, despite expert opinions, no studies in the literature demonstrate that nephrostomy reduces bleeding or other complications (20). Therefore, the "tubeless" approach should be encouraged for future generations of surgeons, as well as high-level studies.

Bleeding remains a significant concern in PNL, with rates exceeding 20% in complex cases (10). Our data show a global transfusion rate of 4.5% across all types of cases during the study period, indicating a notable decrease from 11.5% in 2010 to 2.8% in 2022 (p = 0.009). While surgeons’ learning curve and increased experience likely contribute to this reduction, the primary explanation is the routine implementation of tranexamic acid. Following an initial study by Kumar et al. (21), our group conducted a placebo-controlled randomized trial evaluating the impact of tranexamic acid on transfusion rates in complex PNL cases. This study revealed that the use of tranexamic acid reduced the transfusion rate from 10.4% to 2.2% (relative risk, 0.21, p = 0.033; number-needed-to-treat: 12) without increasing complications or adverse effects, confirming its significant value during PNL (22). Furthermore, we observed an improvement in success rates, potentially due to enhanced surgical visualization. Consequently, a 1g dose of tranexamic acid during anesthetic induction has become standard practice for most PNL cases in our service, and it has undeniably played a crucial role in lowering transfusion rates.

The overall complication rate was 12.2%, showing a decrease from 18.2% in 2009 to 12% in 2022 (p = 0.002). These rates are consistent with those cited in the EAU Guideline on Urolithiasis(1). This reduction can be attributed to several factors introduced over the study period. The learning curve of the surgical team likely played a role, alongside the adoption of key procedural enhancements.

These include:

- Routine antibiotic prophylaxis: Administering nitrofurantoin 7 days before surgery (23, 24).
- Preoperative CT scans for all cases: This allows for better visualization of perirenal organs, significantly reducing the risk of inadvertent puncture, as well as the classification according to the Guy's Stone Score (25).
- Routine use of tranexamic acid: As previously discussed, this has helped manage bleeding.
- Routine use of supine positioning, reducing the surgical time by 30 minutes.
- Ultrasound-guided PCNL: Improving precision during the procedure.
- Regular use of flexible instruments: This allows surgeons to reach parallel calyces, minimizing the need for multiple punctures (26).
- Planned two-stage surgeries: For cases with a large stone burden that cannot be treated in under 120 minutes, this approach helps manage complex situations more effectively.

All these advancements have collectively contributed to the observed improvement in patient outcomes.

The length of hospital stay significantly decreased across both institutions. At HC, the rate of hospitalizations lasting up to 4 days increased from 82% in 2009 to 92.1% in 2022 (p<0.001). Concurrently, at HB, the average length of stay dropped from 81.8 hours (SD ± 69.4) in 2009 to 50 hours (SD ± 25.3) in 2022. Presently, it's not uncommon for patients to undergo surgery in the afternoon and be discharged the following morning, particularly if a tubeless approach was utilized. This mirrors the growing trend of ambulatory surgery, which has been safely performed for select cases, as reported by Du et al. (27). This overall reduction in inpatient time directly reflects a decrease in complications and an increase in the rate of tubeless procedures.

Regarding the success rate, our results are verified by a CT scan performed on the first postoperative day. While we acknowledge that some residual fragments may be expelled up to 90 days post-op, we opted for this early CT for several reasons: to determine our immediate success rate, investigate potential complications, and for logistical convenience, as it's easier to ensure a postoperative CT scan while the patient is still hospitalized. Considering these factors, our overall success rate, defined as the absence of fragments larger than 4 mm on the first postoperative day's CT scan, was 59.8%. The immediate success rates across both services, stratified by the Guy's Stone Score, were 85.7%, 63.7%, 43%, and 28.2% for Guy's 1, 2, 3, and 4 cases, respectively (p<0.001). With auxiliary procedures, our success rate can exceed 90% (28). However, approximately 30% of patients require more than one procedure, which incurs substantial financial and social costs. Having significantly improved the safety of the procedure, we now believe there's room to enhance the immediate success rate. To this end, our ECIRS rate is increasing, a technique shown to improve success(8). The most impactful advancement, we believe, will be the use of intraoperative CT scans to confirm stone-free status. This allows for immediate detection and removal of residual stones, thereby significantly boosting success rates. Although it may seem futuristic, intraoperative CT scanning for this purpose might be closer than we imagine (29).

Our study has some limitations, including its retrospective design and minor differences in protocols between the two institutions, such as the method used to measure inpatient time. However, our reliance on a prospectively maintained electronic database and pre- and postoperative CT scans for all cases ensures the high reliability of our results. We believe this represents the largest series to date, demonstrating the temporal evolution of PNL. It clearly illustrates that this crucial technique is under continuous development and will remain vital for treating complex kidney stones for a long time.

In the early 2000s, PNL procedures were quite different. They were performed with the patient in the prone position, and puncture guidance relied solely on fluoroscopy and findings from excretory urography. This often led to high rates of infectious and hemorrhagic complications. It was routine practice to manually squeeze irrigating fluid to improve visibility in a reddish operative field, and blood transfusion bags were commonly hanging nearby. Surgeries were frequently interrupted and didn't conclude in one go. Patients typically received a nephrostomy tube, were admitted to the ICU, and stayed hospitalized for 3 to 4 days, with surgical success assessed by a simple radiograph.

Fast forward to today: PNL has evolved significantly. A patient's surgery is now meticulously planned based on a CT scan, and their case is classified using Guy's stone score. Patients receive appropriate antibiotic prophylaxis. During the procedure, they are positioned supine, and tranexamic acid is administered to prevent bleeding. Puncture is precisely guided by a combination of fluoroscopy, ultrasonography, and flexible nephroscopy. An ultrasonic lithotripter is regularly used. Surgical success is immediately verified with a flexible ureteroscope. Importantly, patients generally don't require a nephrostomy tube, experience fewer complications, and are often discharged within 24 to 48 hours, with their stone-free status confirmed by a postoperative CT scan. The entire surgical process is now standardized, making surgeons confident in performing PNL. We have truly evolved.

Our analysis of the continuous development of PNL in our departments has led our group to several perceptions about how an ideal PCNL procedure could be performed today or in the near future. In Table-3, we show what we believe to be the state-of-the-art PNL.

**Table 3:** The Ideal PCNL: A Vision for Today and Tomorrow.

**Preoperative Evaluation:** Always conducted with a **CT scan**, allowing classification using a predictive scoring system for success and complications. **Guy's Stone Score** stands out as the most rapid and practical option. For complex cases, volumetric reconstructions, 3D printing of case models, and the use of virtual reality can significantly aid surgical planning.
**Patient Preparation:** Involves adequate preparation of the patient, including **antibiotic prophylaxis** for 7 days before surgery, with nitrofurantoin being an excellent choice.
**Surgical Positioning:** The supine position, in any of its variations, should be the standard. The **Barts Flank Free** position is particularly suitable, as it facilitates combined surgeries and reduces the risk of infectious complications.
**Bleeding Management:** Routine use of **tranexamic acid** is recommended, especially in cases with higher bleeding risk, but potentially even in simpler cases where miniaturized devices are used. It leads to less bleeding and, arguably, better visualization of the operative field, contributing to increased success rates.
**Puncture Guidance: Ultrasound-guided puncture** should gain prominence, combined with endoscopic vision to assist dilation, thereby minimizing the need for fluoroscopy. For complex punctures, intraoperative CT scans can provide invaluable guidance.
**Surgical Technique: Routine ECIRS** should replace conventional PCNL, reserving the latter for cases of urinary diversion or very simple cases. An ultrasonic device is the ideal lithotripter.
**Tract Size:** The pursuit of reduced single tracts is crucial. Complex and bulky cases should be managed with tracts up to **24 Fr** and an ultrasonic lithotripter. Intermediate stone burdens, conversely, can be effectively treated via accesses between 11 and 18 Fr using high-power laser or ultrasonic lithotripters.
**Immediate Success and Ancillary Procedures:** Our next frontier involves utilizing portable tomography devices or existing equipment in hybrid operating rooms at the end of surgery. This aims to improve immediate success rates and reduce the need for ancillary procedures and reapproaches. Despite seeming somewhat futuristic, intraoperative CT scanning may be closer than we imagine.
**Nephrostomy Use:** The use of a nephrostomy tube at the end of surgery could be reserved for **highly selected cases**, such as the presence of pyuria at the puncture site or planned two-stage surgeries where the same access route will be reused for a subsequent approach within a few days. Ureteral drainage with a Double J stent or ureteral catheter remains an option, depending on the specific case.
**Postoperative Pain Management:** Local anesthesia using ropivacaine or bupivacaine, administered from the skin incision to the end of the procedure, effectively reduces postoperative pain and should be routinely performed in all cases. NSAIDs are enough and usually, opioids are not necessary.
**Outpatient PCNL:** With reduced surgical aggression, **outpatient PCNL** can be adopted for some cases. This approach offers substantial benefits by lowering the social and economic impact on society without increasing patient morbidity.
Vicentini FC et al., 2025

In summary, we've clearly observed a significant evolution in PNL outcomes within our departments. There's undeniable evidence of improvement across several key metrics: a reduction in complications, shorter fluoroscopy times, lower nephrostomy rates, and decreased length of hospital stay. While these advancements are substantial, we recognize that there's still room to achieve even higher immediate success rates. We’re actively pursuing this goal through the application of new techniques currently under investigation. Reducing the access caliper may be desirable, too (30). Our ongoing research has been instrumental in refining these parameters and standardizing PNL procedures, making them vastly different from how they were performed before these studies.

## CONCLUSIONS

Percutaneous nephrolithotomy stands as a safe and efficient treatment for large kidney stones. We've documented a clear reduction in complications, transfusions, nephrostomy use rates, and overall length of hospital stay. While significant progress has been made, increasing immediate success rates remains a desirable and achievable objective, driven by the continuous pursuit of technical improvements.

## Data Availability

All data generated or analysed during this study are included in this published article
